# Gut microbiota and dietary intervention: affecting immunotherapy efficacy in non–small cell lung cancer

**DOI:** 10.3389/fimmu.2024.1343450

**Published:** 2024-02-01

**Authors:** Yu Xin, Chen-Guang Liu, Dan Zang, Jun Chen

**Affiliations:** Department of Oncology, The Second Hospital of Dalian Medical University, Dalian, China

**Keywords:** non-small cell lung cancer, gut microbiota, PD-1/PD-L1, antibiotics, dietary intervention

## Abstract

Non–small cell lung cancer (NSCLC) accounts for 80–85% of all lung cancers. In recent years, treatment with immune checkpoint inhibitors (ICIs) has gradually improved the survival rate of patients with NSCLC, especially those in the advanced stages. ICIs can block the tolerance pathways that are overexpressed by tumor cells and maintain the protective activity of immune system components against cancer cells. Emerging clinical evidence suggests that gut microbiota may modulate responses to ICIs treatment, possibly holding a key role in tumor immune surveillance and the efficacy of ICIs. Studies have also shown that diet can influence the abundance of gut microbiota in humans, therefore, dietary interventions and the adjustment of the gut microbiota is a novel and promising treatment strategy for adjunctive cancer therapy. This review comprehensively summarizes the effects of gut microbiota, antibiotics (ATBs), and dietary intervention on the efficacy of immunotherapy in NSCLC, with the aim of informing the development of novel strategies in NSCLC immunotherapy.

## 1 Introduction

Lung cancer currently has an extremely high incidence and mortality rate, with non–small cell lung cancer (NSCLC) accounting for 80–85% of all lung cancers. Therefore, clarifying the pathogenesis and treatment of NSCLC is a critical healthcare need. The programmed cell death 1 (PD-1) and programmed cell death ligand 1 (PD-L1) axis play a key role in physiological immune homeostasis and can serve as a means for cancer cells to evade the immune system (1). PD-L1 binds its major receptor PD-1 in trans and cis (2, 3) by antagonizing T-cell receptors and CD28 costimulatory signals (4) to suppress antitumor PD-1–positive T-cell function (5). The PD-1 axis also inhibits the lytic activity against activated cells, including B cells and natural killer (NK) cells (6, 7). More importantly, PD-1 is highly expressed in regulatory T cells (Tregs), which can be activated and proliferated in the presence of the ligand (8). It can also suppress the immune response through increasing the expression of forkhead transcription factor FOXP3, muting the expression of effector cytokines such as interferon-gama (IFN-γ), and producing inhibitory cytokines such as transforming growth factor-beta (TGF-β), interleukin (IL)-10, and IL-35. Anti–PD-1/PD-L1 therapy is an effective treatment for patients with metastatic NSCLC lacking sensitizing EGFR or ALK mutations (9–13). The primary anticancer mechanism of anti–PD-L1 or anti-PD-1 antibody is thought to prevent PD-1 antitumor T cells from being inhibited by cell surface–expressed PD-L1, leading to T-cell resuscitation (14), reduced T-cell depletion or death, and increased T-cell memory and intratumoral antitumor immune cell infiltration (15–19). PD-L1 expressed on tumor cells is considered to be a biomarker for predicting immune checkpoint inhibitors (ICIs) efficacy in patients with NSCLC (20).

Overall and objective response rates improve significantly in some patients after immunotherapy (21). However, due to individual differences among patients (22), only a minority of patients show benefit from ICIs (23). Emerging evidence indicates that the efficacy of ICIs therapy is related to the characteristics of the host gut microbiota (24, 25). The gut microbiota is being increasingly recognized as an important factor associated with tumor development and efficacy of antitumor therapies (26). Various studies have confirmed that the gut microbiota can significantly influence ICIs treatment (27–30), and research indicates that complete gut microbiota is essential to improving the efficacy of ICIs and cancer treatment (27, 31–33). Patients with a favorable gut microbiota show enhanced memory T cell and NK cell signatures in the peripheral blood (34). The gut microbiota is emerging as an attractive therapeutic target for cancer. Using antibiotics (ATBs) has been shown to impair the efficacy of immunotherapy in mice and human (27, 31), an effect which may be attributable to a dramatic reduction in gut dysbiosis and beneficial microbial subpopulations. Therefore, it is important to strictly control the use of ATBs during immunotherapy in patients with tumor.

In addition, it has been proven that dietary intervention can regulate gut microbiota and affect the efficacy of immunotherapy. Studies have shown that probiotics use is associated with favorable clinical outcomes in patients with advanced or recurrent NSCLC receiving anti-PD-1 monotherapy. In patients with NSCLC receiving ICIs, combining probiotic therapy has been reported to result in significantly longer progression-free survival (PFS) and overall survival (OS) (35–37). Reasonable use of probiotics, prebiotics, and dietary intervention to target the gut microbiota may be a potential strategy for promoting the clinical efficacy of ICI treatment (38). Although the gastrointestinal tract and respiratory tract are physically distant, they share the same embryonic origin, and they are highly similar structurally (39). Recent findings attesting to the many pathways involving their respective microbiota support the presence of the gut–lung axis (GLA) (40), suggesting that gut microbiota can influence the development of lung cancer.

**Figure 1:**
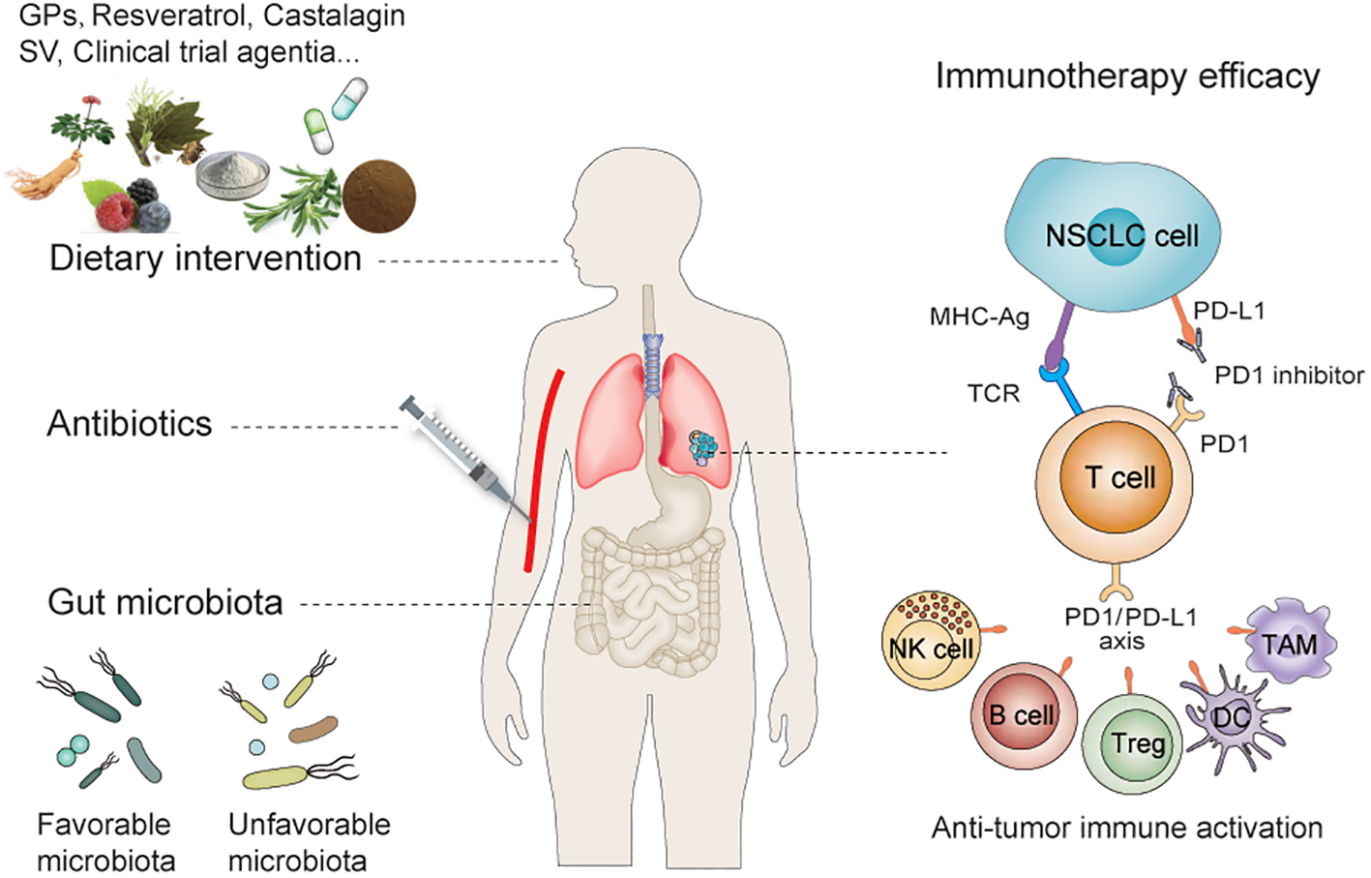
Dietary intervention and gut microbiota regulate the efficacy of immunotherapy for NSCLC. Dietary interventions and antibiotics can influence the efficacy of anti-PD-1/PD-L1 treatment by influencing the abundance of gut microbiota—which includes the synergistic and antagonistic effect of microbiota on PD-1/PD-L1 therapy—by modulating innate and/or adaptive immunity. NSCLC, Non–small Cell Lung Cancer; PD-L1, Programmed cell death ligand 1; PD-1, Programmed cell death 1.

In this review, we focus on the “favorable” and “unfavorable” gut microbiota in preclinical and clinical studies related to NSCLC immunotherapy, particularly anti-PD-1/PD-L1 therapy, and the impact of ATBs and dietary interventions on the efficacy of NSCLC immunotherapy (**Figure 1**).

## 2 Gut microbiota associated with NSCLC immunotherapy efficacy

The complex gut microbial ecosystem plays a role in human health and disease, and recent evidence suggests that the microbiota is linked to the efficacy and toxicity of different cancer treatments (41). The gut microbiota has emerged as a key factor in shaping tumor immune surveillance and determining the efficacy of ICIs (**Table 1**).

**Table 1:** Gut microbiota targeting innate and adaptive immune cells to affect the efficacy of anti-PD-1/PD-L1 treatment in NSCLC.

Gut microbiota		Relationship between gut microbiota and ICIs	Interventions factors and/or biological effects	ICIs	Reference
*Bifidobacterium*	B.HIF-K57, B.HIF-K18	Synergistic	T cells	PD-1	(29)
	B.HIF-MG731 (B.HIF-M31)	Synergistic	T cells	PD-1	(29)
	Bifidobacterium longum	Synergistic	INF-a	PD-1	(42, 43)
	Bifidobacterium breve	Synergistic	H2-kb SIY	PD-1	(43, 44)
*Lactobacillus rhamnosus*		Synergistic	________	PD-1	(45–47)
*Akkermansia muciniphila*		Synergistic	CCR9+, CXCR 3+, CD4+ T lymphocytes, HYR-2	PD-1/PD-L1	(27, 29, 48–53)
*Firmicutes and actinomyces*		Synergistic	________	PD-1	(54)
*Enterococcus bacteriophage*		Synergistic	CD8+ T lymphocytes	PD-1	(55)
*Granulicatella*		Synergistic	________	PD-1	(56)
*Alistipes*		Synergistic	________	PD-1	(27, 34, 51, 57)
*Escherichia coli* Nissle 1917		Synergistic	CD4+ T lymphocytes, CD8+ T lymphocytes	PD-L1	(58)
Gammaproteobacteria		Antagonistic	________	PD-L1	(59)
*Helicobacter pylori*		Antagonistic	DCs, CD8+ T lymphocytes	PD-1	(60)

PD-1, programmed cell death 1; PD-L1, programmed cell death ligand 1; NSCLC, non–small cell lung cancer; ICIs, immune checkpoint inhibitors.

### 2.1 Favorable gut microbiota

**Figure 2:**
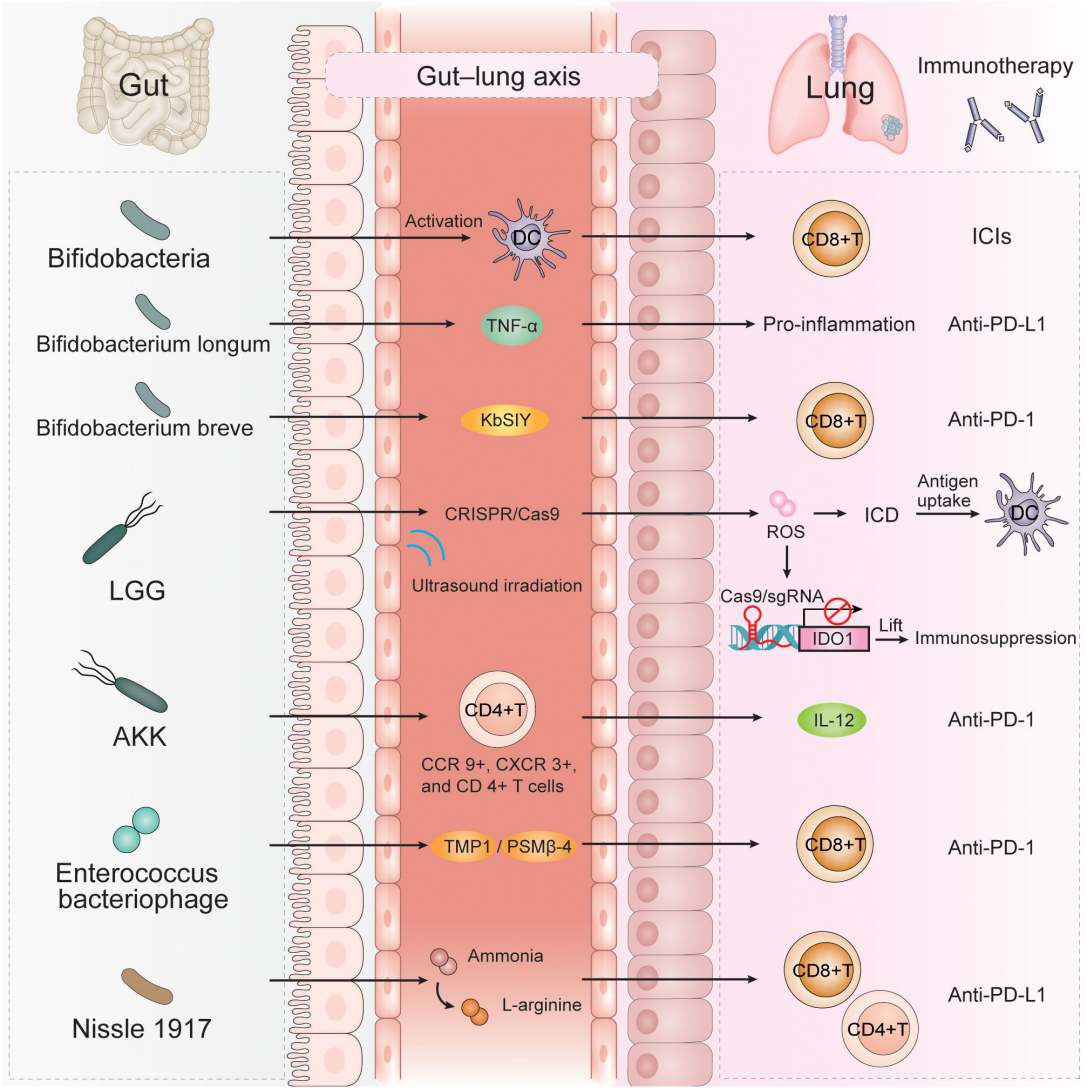
A variety of gut microbiota can affect the efficacy of immunotherapy for NSCLC through multiple immune mechanisms, such as *Bifidobacteria*, *Bifidobacterium longum*, *Bifidobacterium breve*, LGG, AKK, *Enterococcus bacteriophage*, *Nissle 1917.* AKK, *Akkermansia muciniphila*; LGG, *Lactobacillus rhamnosus GG;* TNFα, tumor necrosis factor alpha; KbSIY, a model neoantigen, SIYRYYGL; CRISPR/Cas9, clustered regularly interspaced short palindromic repeat; ROS, reactive oxygen species; ICD, immunogenic cell death; IDO1, indolea-mine 2,3-dioxygenase-1; TMP1, the epitope-tail length measurement protein 1; PSMβ-4, proteasome subunit beta-4.

The human gut contains certain probiotics, and rational use of anti-PD-1/PD-L1 therapy combined with probiotics can significantly improve the efficacy of anti-PD-1/PD-L1 therapy in NSCLC, providing more opportunities for the prolonging of PFS and OS in those with NSCLC (**Figure 2**).

#### 2.1.1 Bifidobacteria

Bifidobacteria are key intestinal beneficial microorganisms, which as a type of physiologically beneficial bacteria, can exert an antitumor effect. Bifidobacterium species have been shown to be strongly associated with ICIs efficacy (57, 61), mainly by promoting the activation of dendritic cells (DCs) under homeostasis, which in turn improves the effector function of tumor-specific CD8+ T cells (29). Sivan (29) et al. showed that the therapeutic effect of Bifidobacterium was abolished in mice lacking CD8+ T cells, suggesting that the mechanism was not direct but exerted through the host antitumor T-cell response. Only specific Bifidobacterium strains (i.e., B. HIF-K57, B. HIF-K18, and B. HIF-MG731 [B. HIF-M31]) act synergistically with anti–PD-1 therapy to reduce tumor growth, while other Bifidobacterium bifidum strains (i.e., B. bifidum_B06, B. bifidum_R71 and B. bifidum CKDB001 (B. Bifidum_C01)) show no synergistic effect with anti-PD-1 therapy (62). It can be surmised that even different strains of the same bacteria have different effects on the efficacy of immunotherapy. Wu et al. (42) showed that Bifidobacterium longum (B. longum) had exert an antitumor effect but also had an antagonistic effect against pembrolizumab treatment. In addition, flow cytometry showed that the proportion of CD45+ cells in peripheral blood significantly changes after B. longum treatment (42). Another study found that in a mouse model of B16.SIY tumor, B. longum increased production of proinflammatory cytokine tumor necrosis factor alpha (TNFα) and significantly improved tumor control after anti-PD-L1 treatment (43). Moreover, studies have reported that Bifidobacterium breve (B. breve) in the gut significantly prolong the median PFS (mPFS) of patients with NSCLC receiving anti–PD-1 immunotherapy combined with chemotherapy, and B. breve may be an effective biomarker for predicting its clinical benefit (44). B. breve exposure can promote the ability of H2-Kb SIY (a model neoantigen, SIYRYYGL) (KbSIY) complex reactive cells to expand, resulting in an increase in CD8+ T cells, a higher affinity response, and greater KbSIY cross-reactivity, with targeted KbSVY (an epitope called SVYRYYGL) therapy being shown to slow tumor progression (43). These findings suggest that bifidobacteria are beneficial for antitumor immune responses. Despite the above mentioned findings, further identification of the effects of different Bifidobacterium strains on immunotherapy and their molecular mechanisms remains a challenge that requires continued research.

#### 2.1.2 Lactobacillus rhamnosus

Lactobacillus rhamnosus GG (LGG), a species belonging to the Lactobacillus genus, is a Gram-positive, anaerobic, inactive, non–spore-forming, rod-shaped microbe that is one of the most widely studied probiotics in humans. LGG can penetrate the hypoxia tumor center, allowing efficient delivery of the clustered regularly interspaced short palindromic repeat (CRISPR/Cas9) system to the tumor region. The CRISPR/Cas9 nanosystem can generate abundant reactive oxygen species (ROS) under the ultrasound irradiation, resulting in immunogenic cell death (ICD), while the produced ROS can induce endosomal/lysosomal rupture and then releasing Cas9/sgRNA to knock down the indolea-mine 2,3-dioxygenase-1 (IDO1) gene to lift immunosuppression (45). Recent studies have found that Lactobacillus rhamnosus Probio-M9 can effectively restore the diversity and structure of intestinal microbes in mice, but ProbioM9 alone has no significant effect on tumor inhibition. However, in anti-PD-1 therapy, Probio-M9 inhibits harmful bacteria by enhancing beneficial bacteria, thus promoting an antitumor immune response to anti-PD-1 therapy (46, 47).

#### 2.1.3 Akkermansia muciniphila

Akkermansia muciniphila (AKK) is a Gram-negative and anaerobic bacterium that is a minor component of the gut microbiota. It is generally acquired from the mother, is usually abundant in children, and decreases with age and disease. The relative abundance of AKK has been associated with human responses to anti-PD-1 or anti-PD-L1 therapy (29, 48, 63). Patients with AKK in the gut respond better to treatment (49). And in the NEOSTAR trial, Neoadjuvant ipilimumab+nivolumab (Ipi+Nivo) and nivolumab+chemotherapy (Nivo+CT) induce greater major pathologic response (MPR) rates than CT alone in patients with operable NSCLC. MPR rates were 32.1% in the Nivo + CT arm and 50% in the Ipi+Nivo+CT arm. Although the MPR was different between the two groups, both groups were AKK-rich in the fecal microbiota at baseline (64). However, the abundance of AKK is reduced in PD-1 nonresponders (50). Hypermucotropic AKK has been associated with durable clinical benefit in patients with advanced lung cancer (51).

The proportion of AKK is higher in patients with stable disease and partial response to immunotherapy compared with patients with progressive disease (49, 52). In one study, the objective response rate (ORR) was greater in AKK-positive patients than in AKK-negative patients receiving immunotherapy alone in first-line treatment. Moreover, 59% percent of the AKK-positive patients were still alive after 12 months, whereas only 35% of AKK-negative individuals were long-term survivors. For the entire cohort of patients (regardless of treatment regimen), the median OS (mOS) in the AKK-positive group was greater than in the AKK-negative group (63).

Routy et al. showed that oral supplementation with AKK in nonresponders restored the efficacy of PD-1 blockade in an IL-12-dependent manner by increasing the recruitment of CCR9+, CXCR3+, and CD4+ T lymphocytes to tumor beds in mice (27). Teng et al. reported that HYR-2, which is composed of Salvia miltiorrhiza, ginseng, and licorice at half the dose of the Ze Qi decoction, may exert its anti–lung cancer effect by downregulating PD-L1 and upregulating AKK mucotrophism. HYR-2 may down-regulate PD-L1 expression *in vivo* by inhibiting the PI3K/Akt signaling pathway (53). Thus, AKK can be used as a supportive marker of response to immunotherapy in NSCLC.

#### 2.1.4 Other gut microbiota

Evidence from clinical trials suggests that *Firmicutes* and *Actinomyces* are enriched in responders receiving intestinal flora transplantation (FMT) combined with PD-1 blockade (54). The epitope-tail length measurement protein 1 (TMP1) in the genome of *Enterococcus* bacteriophage is highly similar to the proteasome subunit beta-4 (PSMβ**-**4) tumor antigen. They can simultaneously activate CD8+ T cells and improve the efficacy of PD-1 blockade (55). Two butyrate-producing gut bacteria (Agathobacter M104/1 and Blautia SR1/5) were found to favorably regulate the host immune response, and the presence of these 2 substances in the stool could significantly prolong PFS (65). In one study, *Granulicatella* showed a significant association with responders to anti-PD-1 therapy and thus could be used as a biomarker for prognosis and diagnosis (56). Other studies have shown that the presence of *Alistipes* is related to better results in ICIs treatment (57), and *Alistipes putredinis* is reduced in patients with advanced NSCLC who respond to anti-PD-1 immunotherapy (34). Moreover, the overexpression of *Alistipes indistinctus* in NSCLC responders was found to be effective in restoring the efficacy of ICIs therapy (27). A high level of *Alistipes onderdonkii* has been associated with durable clinical benefit (DCB) (51). The engineered probiotic *Escherichia coli* Nissle 1917 strain can colonize the tumor site and continuously convert the metabolite ammonia to L-arginine in the tumor bed. Intertumoral injection of this strain in mice was shown to increase intracellular L-arginine concentrations, trigger intratumoral CD4+ and CD8+ T-cell infiltration, and exert a synergistic antitumor effect when combined with anti-PD-L1 therapy (58).

### 2.2 Unfavorable gut microbiota

Not all gut microbiota play an auxiliary role in anti-PD-1/PD-L1 therapy in NSCLC, and some gut microbiota have been shown to be associated with poor response to ICIs treatment and poor survival.

#### 2.2.1 Gammaproteobacteria

Gammaproteobacteria are the most diverse Gram-negative bacteria. The presence of Gammaproteobacteria is associated with low PD-L1 expression and poor response to checkpoint-based immunotherapy, resulting in poor survival (59). Gammaproteobacteria appear to be enriched in lung cancer, and their abundance in the tumor environment is associated with low PD-L1 expression and a tendency for low PFS and worse OS in ICIs therapy (59).

#### 2.2.2 Helicobacter pylori

Helicobacter pylori is a typical spiral or arc bacteria located on the surface of gastric mucosal epithelial cells, appearing mainly on the mucosa near the pylorus and antrum of the stomach. In the Dijon cohort study, H. pylori seropositivity was found to be associated with reduced survival in patients with NSCLC treated with anti-PD-1 therapy. H. pylori–seropositive patients had a median survival of 6.7 months, compared with 15.4 months for seronegative patients. In addition, H. pylori seropositivity was found to be associated with significantly reduced PFS in patients with NSCLC cancer treated with anti-PD-1 therapy (60). H. pylori inhibits antitumor CD8+ T-cell responses by altering the cross-presenting activity of DCs and inhibits both innate and adaptive immune responses in infected hosts. Although H. pylori does not colonize the gut, based on the data we have available, it is critical to eradicate it when possible during anti-PD-1/PD-L1 therapy in patients with NSCLC. It has been discovered that the gut microbiota is a key factor affecting the efficacy of ICIs in NSCLC and can be used as a biomarker to predict the efficacy of ICIs treatment. However, the specific mechanism of action needs to be further studied. It is expected that other clinically significant intestinal flora will be discovered to provide a basis for targeting the advantaged population in NSCLC immunotherapy.

## 3 Influence of ATBs on immunotherapy efficacy in patients with NSCLC

There are conflicting opinions concerning the effect of ATBs use on ICIs treatment. It has been shown that ATBs use is an independent predictor of shorter PFS and OS in patients with advanced cancer treated with ICIs (31, 66–72). In addition, several meta-analyses also demonstrated that ATB use was significantly associated with adverse OS and PFS in cancer patients receiving ICI immunotherapy (33, 73, 74). This negative antitumor effect is significantly enhanced with the cumulative use of ATBs (66). Schett et al. reported that patients with NSCLC who received ATBs within 60 days before ICIs had significantly shorter PFS and OS than did patients who did not receive ATBs before ICIs (70). Other studies indicate that at 60 days before ICIs initiation, ATBs have no effect in terms of objective response or PFS but may still be significantly associated with shorter OS (31, 75). Furthermore, the recent study indicated that the downregulation of mucosal addressin cell adhesion molecule 1 (MAdCAM-1) in the ileum, following ATBs treatment, led to gut recolonization by Enterocloster species. This, in turn, induced the emigration of enterotropic α4β7+CD4+ regulatory T 17 cells into the tumor. In independent cohorts of lung cancer patients, it was observed that low serum levels of soluble MAdCAM-1 had an adverse prognostic impact (76).

However, other studies did not find any difference in PFS and OS between patients treated with ATBs and those without treatment. Galli et al. propose that the duration of ATBs use associated with immunotherapy may be the most relevant factor in modulating PFS and OS, and not the type of ATBs prescribed or the time between the start of ATBs use and the start of immunotherapy. In their study, patients with high ATBs–immunotherapy exposure ratio (AIER) during the full immunotherapy period had worse PFS and OS compared to other patients (77). Previous studies have proposed a measure called ATBs exposure (AE), which is defined as the number of ATBs days divided by ICIs days. In a comparison of patients who received ATBs treatment and those who did not within a 4-week period before or after starting ICIs therapy, there were no differences in PFS or OS. However, it was observed that patients with higher AE had a significantly lower PFS and OS than did those with a median AE. This suggests that the cumulative use of ATBs, rather than its use within a specific time frame, may negatively impact the effectiveness of immunotherapy (78). Kaderbhai et al. conducted a study on patients with NSCLC and found that ATB-induced changes in the microbiota did not seem to affect the efficacy of nivolumab in terms of PFS (79).

Regarding the type of ATBs used, Lu et al. reported that that patients receiving fluoroquinolones exhibited worse OS than did those treated with other ATBs, and patients who received beta-lactamase inhibitors (BLBLI), fluoroquinolones, or sulfonamides 30 or 60 days prior to ICIs treatment had shorter PFS and OS. In addition, the effect of ATBs use during the first 60 days of ICIs therapy was less pronounced than during the first 30 days (72). In a study by Kim et al., patients treated with BLBLI showed a tendency toward longer PFS and OS compared to patients treated with other types of ATBs (68). Although there are differences between the results of the studies, we can see that the use of most ATBs is inversely related to the efficacy of immunotherapy. Therefore, it is clinically recommended that the use of ATBs should be strictly controlled during immunotherapy in patients with cancer.

## 4 Effect of dietary intervention on the efficacy of immunotherapy in NSCLC

**Figure 3:**
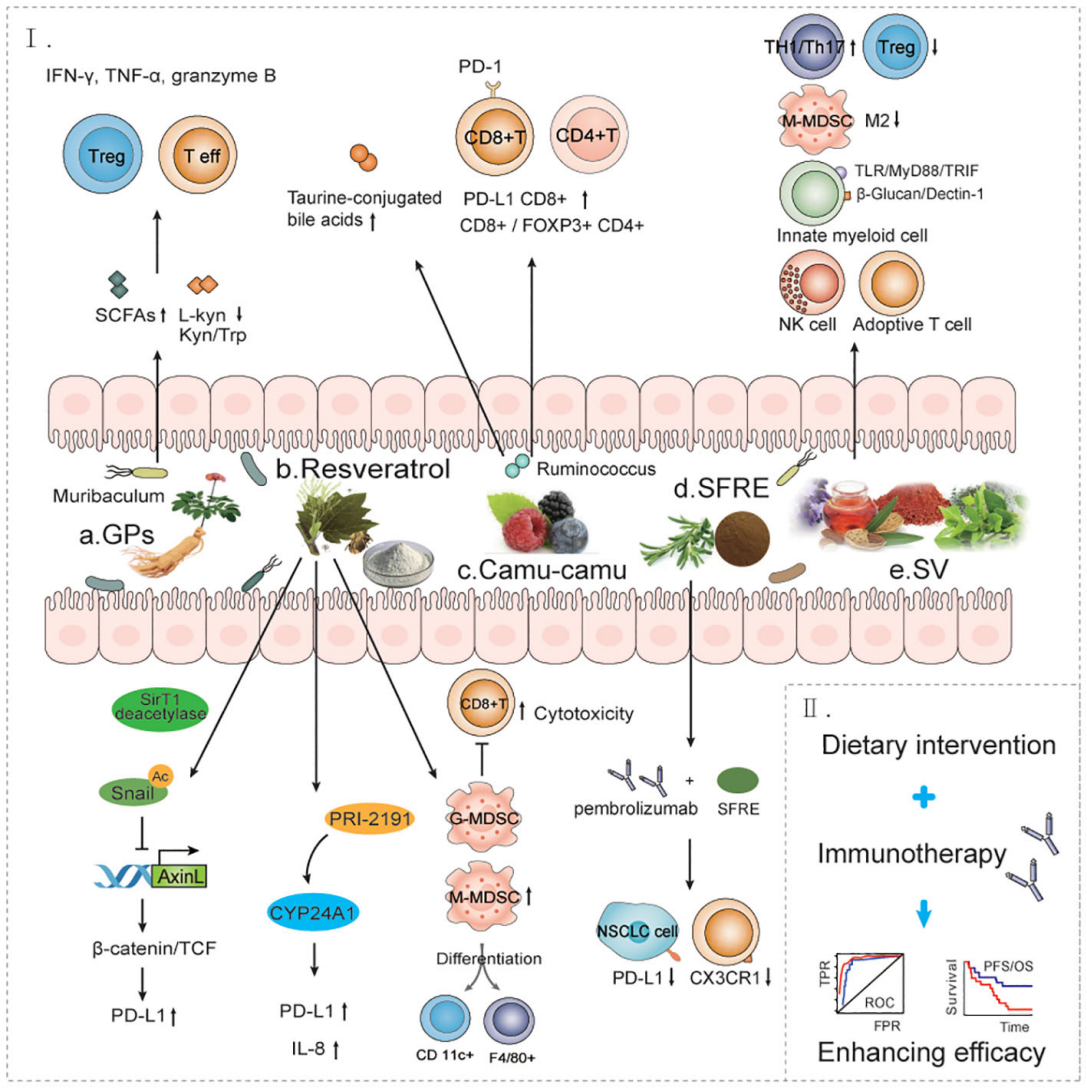
The effect of dietary interventions for the treatment of NSCLC immune. a. GPs increases the antitumor response to anti-PD-1 antibodies by regulating bacteria such as Muribaculum. b. RESV can improve the tumor immunosuppressive microenvironment through multiple mechanisms and promote immune cell-mediated immunotherapy response. c. Castalagin increases the anti-tumor response of anti-PD-1 antibodies by modulating bacteria such as Ruminococcus. d. SFRE combined with pembrolizumab enhanced the anti-PD-L1 immune response of tumor cells and improved the efficacy of immunotherapy. e. SV improves the immunosuppressive microenvironment of tumors by promoting multiple immune response pathways. f. Dietary intervention combined with immunotherapy improves immunotherapy efficacy and prolongs overall survival in patients with NSCLC. GPs, Ginseng polysaccharides; RESV, Resveratrol; SFRE, Supercritical extraction of rosemary; SV, Specific vegetable extracts.

Dietary intervention or prebiotics supplementation for patients with NSCLC receiving anti-PD-1/PD-L1 therapy may be a more promising approach for the general population, as it is not only less harmful but also more tolerable. Studies have shown that diet can alter gut microbiota abundance in humans, with short-term consumption of diets consisting exclusively of animal or plant products altering interindividual differences in microbial community structure and microbial gene expression (80). Dietary supplements may influence the gut microbiota and response to anti-PD-1 immunotherapy (**Figure 3**).

### 4.1 Ginseng polysaccharides

Ginseng polysaccharides (GPs) are one of the most abundant components in ginseng, which can exert key immunomodulatory and antitumor effects. One study found that GPs increased the antitumor response to anti-PD-1 monoclonal antibodies by increasing the microbial metabolite valine and decreasing the ratio of L-kynuridine and the ratio of kynurenine to tryptophan, which helped suppress Tregs and induced effector T cells after combination therapy. Combination therapy with GPs and anti-PD-1 monoclonal antibodies reshaped the composition of the gut microbiota and increased the amount of short-chain fatty-acid–producing bacteria, Muribaculum, to sensitize the antitumor effect of anti-PD-1 therapy, restoring the composition of microbes in fecal samples from nonresponders to anti-PD-1 therapy. In another study, combination therapy increased the production of functional cytokines IFNγ, TNFα, and granzyme B by CD8+ T cells in peripheral blood and tumor tissue (80). These data suggest that combination therapy enhances the antitumor immunity effect.

### 4.2 Resveratrol

Resveratrol (RESV), a nonflavonoid polyphenol organic compound, is an antitoxin produced by many plants when stimulated. The pharmacology of RESV in lung cancer cells enables dose-dependent upregulation of PD-L1 expression in the concentration range and is critical for suppressing T cell–mediated immune responses. Furthermore, the Wnt pathway mediates the upregulation of PD-L1 induced by RESV. Mechanistically, RESV activates Sirt 1 deacetylase to deacetylate and stabilize transcription factor Snail. Snail in turn represses Axin 2 transcription, which leads to disassembly of the destruction complex and enhanced binding of β-catenin/TCF to the PD-L1 promoter (81). In addition, RESV may affect vitamin D signaling in lung cancer cells. The vitamin D–active metabolite PRI-2191 was shown to cause a significant upregulation of PD-L1 expression in HCC 827 and NCI-H358 cells, and only when PRI-2191 was used with RESV was PD-L1 expression significantly increased (82). RESV can upregulate the cytotoxic effect of CD8+ T cells and improve the tumor immunosuppressive microenvironment, and the combination of RESV with other immunotherapy drugs may be a more effective treatment (83). RESV is considered to be a module of the inhibitory function of medullary-derived suppressor cells (MDSC), a novel booster of tumor immunotherapy. RESV has been shown to ameliorate tumor development by decreasing granulocytic MDSC (G‐MDSC) accumulation, impairing its suppressive ability on CD8+T cells and promoting monocytic MDSC (M‐MDSC) differentiation into CD11c+ and F4/80+ cells (84).

### 4.3 Polyphenols

#### 4.3.1 Castalagin

Castalagin is a polyphenol that enhances resistance to PD-1. In their study, Messaoudene et al. reported that oral supplementation with polyphenol-rich berry camu-camu (CC; Myrciaria dubia) altered the gut microbial composition, leading to antitumor activity and a stronger anti-PD-1 response. Castalagin improved the CD8+/FOXP3+CD4+ ratio in the tumor microenvironment. Moreover, castalagin induced metabolic changes, resulting in an increase level of taurine-conjugated bile acids. Ruminococcus-rich NSCLC responders were found to be able to metabolize castalagin. The increase in Ruminococcus after cancer treatment further suggests that castalagin has a propensity to interact with specific and beneficial bacteria (85).

#### 4.3.2 Supercritical extraction of rosemary

Supercritical extraction of rosemary (SFRE) (12-16% composition of phenolic diterpenoid, carnosic acid, and carnol) works synergically with the standard treatment used in the clinic to inhibit the cell viability of NSCLC cells. SFRE was found to reduce the expression of PD-L1 and CX3CRl, which exert immunosuppressive effects in the tumor microenvironment, suggesting that SFRE may contribute to the reduction of immune evasion by lung cancer cells (86).

### 4.4 Specific vegetable extracts

Specific vegetable extracts (SVs) have been shown to benefit the survival of patients with stage IIIb/IV NSCLC, and SV can regulate the antitumor efficacy of NK and adoptive T-cell immune responses. The antitumor effects of SV are also mediated by innate bone marrow cell function, which requires toll like receptor (TLR) and β-glucan signaling in a MyD88/TRIF– and Dectin-1–dependent manner, respectively. Furthermore, SV treatment reduced granulocytic MDSC infiltration into the tumor and limited monocytic MDSC toward the M2-like functional phenotype. SV treatment enhances antigen-specific immune responses by enhancing the activation and proliferation of antigen-specific T helper 1 (TH1)/TH 17 cells in secondary lymphoid organs and by reducing the Treg population in the tumor microenvironment. One study found that SVs mainly composed of mushrooms and green bean extract used alone or in combination with ICIs could exert significant antitumor effects in mice with lung cancer (87).

### 4.5 Other dietary interventions

Studies have shown that a sugar-restricted diet improves the host pulmonary immune response and inhibits the tumor growth of experimental lung adenocarcinoma (LUAD). Thus, sugar-restricted diets have become a therapeutic approach for patients with LUAD (88). Methionine regulates tumor immunity by regulating the activity of cyclic GMP-AMP synthase (cGAS), so the tumor immune response can be improved by controlling dietary methionine intake (89). Patients should be advised to minimize their intake of animal meat and increase their intake of plants as much as possible, aiming for 30 plants per week. High fiber intake (>30 g/day) is thought to increase the chance of an ICIs response (90). In addition, alcohol intake has been shown to cause CD4+ T-lymphocyte depletion, allowing for *in situ* growth of LUAD xenografts in BALB/c mice. In addition, alcohol consumption reduces the ability of the compromised immune system to reject tumors. High intake of ethanol delays the recovery of the immune response after CD4+ T-lymphocyte depletion (91).

### 4.6 Clinical trials on dietary and probiotics interventions

In recent years, a number of clinical trials have been carried out examining the efficacy of immunotherapy combined with dietary intervention. If dietary intervention is proven to have a positive auxiliary effect on immunotherapy, this will represent an unprecedented breakthrough in the treatment of lung cancer. One trial (NCT03700437) is investigating a plant-based fasting-mimicking diet (FMD) that provides ~300 calories/fasting day and includes all the food to be consumed during the dietary intervention, including supplements. Participants will start the diet 3 days prior to chemoimmunotherapy and continue on the first day of chemoimmunotherapy for the first 4 cycles of therapy. The aim of this study is to investigate the effect of FMD therapy on chemotherapy combined with immunotherapy for NSCLC. Another trial (NCT04924374) included an experimental group that pooled fecal microbiota capsules of 1 donor selected based on fecal abundance in Faecalibacterium prausnitzii, Bifidobacterium longum, AKK, and Fusobacterium spp. After the screening and metagenomic analysis of 10 donors with high-fiber diets (>30g/day) is performed, anti-PD-1 therapy will be administered every 1–2 weeks. In contrast, the control group will be only administered anti–PD-1 therapy every 2–3 weeks. The aim of this study is to investigate the effect of gut microbiota in patients with high-fiber diet on the efficacy of immunotherapy in patients with advanced NSCLC. In another study (NCT04175769), the experimental group will consist of participants who will orally consume 2 gelatin capsules of nutritional supplement (1 gram), 2 times per day with meals, and 1 capsule 1 additional meal or snack, totaling 5 capsules per day beginning the first day of treatment and continuing for the duration of their treatment. The control group will orally consume 2 gelatin capsules of placebo (1 gram), 2 times per day with meals, and 1 capsule at 1 additional meal or snack, totaling 5 capsules per day beginning the first day of treatment and continuing for the duration of treatment. The aim of this study is to investigate the effect of nutritional products on the response of patients to immunotherapy or immunotherapy combined with chemotherapy for NSCLC. Another trial (NCT05902260) will test whether high-energy and high-protein nutritional supplements can decrease protein clearance, including drug clearance in patients with NSCLC receiving anti-PD-1 ICIs; this in turn would positively affect anti-PD-1 drug bioavailability, leading to activation of the immune system and thereby an increased response to PD-1 therapy. Patients will start with the daily nutritional intervention prior to start of the first infusion of anti-PD-1 therapy (consuming two 200-mL bottles of study product per day) and will continue this nutritional support for 4 treatment cycles, corresponding with 12 weeks of treatment. Blood samples, questionnaires and fecal specimens will be collected on several time points during this treatment. The aim of this study is to investigate the effect of high-energy and high-protein nutritional supplements on anti-PD-1 therapy for NSCLC. In addition, ongoing clinical trials are investigating the correlation between probiotics and immunotherapy. These clinical trials involve a substantial number of participants, with well-designed and representative experimental and control groups. Upon reaching a conclusion that dietary intervention can affect immunotherapy, it will hold significant value in guiding clinical practices. For the latest and more detailed information, please refer to the clinicaltrials.gov database (**Table 2**).

**Table 2:** Ongoing clinical trials designed to investigate the effect of dietary and probiotics interventions on immunotherapy for NSCLC.

NCT number	Patient (n)	Study Title	Intervention	Research purpose	Enrollment status
NCT03700437	12	Fasting-mimicking Diet With Chemo-immunotherapy in Non-small Cell Lung Cancer (NSCLC).	Fasting-mimicking diet	Evaluate the effect of the fasting-mimicking diet therapy on chemotherapy combined with immunotherapy in NSCLC.	Completed
NCT04924374	20	Microbiota Transplant in Advanced Lung Cancer Treated With Immunotherapy.	High-fiber diets	Evaluate the effect of gut microbiota in patients with a high-fiber diet on the efficacy of immunotherapy in patients with advanced NSCLC.	Recruiting
NCT04175769	60	A Nutritional Supplement to Support People With Non-small Cell Lung Cancer.	Nutritional supplement	Evaluate the effect of nutritional products on the response of patients to immunotherapy or immunotherapy combined with chemotherapy in NSCLC.	Recruiting
NCT05902260	50	The Effects of an Nutritional Intervention on PD-1 ICI in NSCLC.	Nutritional intervention	Evaluate the effect of high-energy/high-protein nutritional supplements on anti–PD-1 therapy for NSCLC.	Recruiting
NCT05384873	180	Immunonutrition for Improving the Efficacy of Immunotherapy in Patients With Metastatic Non-small Cell Lung Cancer.	High-calorie-high-protein nutritional liquid supplement enriched in immunonutrients	Evaluate the efficacy of the early systematic provision of oral nutritional supplements enriched in immunonutrients in NSCLC patients undergoing immunotherapy and receiving nutritional counseling.	Recruiting
NCT05865730	122	A Study of Oncobax^®^-AK in Patients With Advanced Solid Tumors.	Live Bacterial Product - Akkermansia muciniphila (Oncobax^®^-AK)	Prove that the oral administration of Oncobax^®^-AK to cancer patients under immunotherapy, but whose gut microbiota is deficient in Akkermansia will restore/improve the efficacy of immunotherapy in patients with NSCLC or RCC.	Recruiting
NCT05303493	45	Camu-Camu Prebiotic and Immune Checkpoint Inhibition in Patients With Non-small Cell Lung Cancer and Melanoma.	Camu Camu Capsules (Camu Camu powder encapsulated (500mg each)	Assess the safety and tolerability of Camu Camu prebiotic in patients with advanced NSCLC and melanoma in combination with standard-of-care ICI.	Recruiting
NCT04699721	40	Clinical Study of Neoadjuvant Chemotherapy and Immunotherapy Combined With Probiotics in Patients With Potential/Resectable NSCLC.	Bifidobacterium trifidum live powder	Evaluate the safety and effect of neoadjuvant chemotherapy and immunotherapy combined with probiotics for early resectable NSCLC patients.	Active, not recruiting
NCT05094167	46	Lactobacillus Bifidobacterium V9 (Kex02) Improving the Efficacy of Carilizumab Combined With Platinum in Non-small Cell Lung Cancer Patients.	Lactobacillus Bifidobacterium V9	Evaluate the efficacy of probiotics Bifidobacterium Lactobacillus V9 (Kex02) in the treatment of NSCLC with lizumab combined with platinum.	Recruiting

PD-1, programmed cell death 1; NSCLC, non–small cell lung cancer.

In recent years, trials on dietary intervention have been carried out in succession. Although many trials are still ongoing, it can be clearly seen that researchers are beginning to turn their attention to the combination of dietary intervention and immunotherapy for NSCLC treatment in order to maximize the efficacy of immunotherapy and maximize the benefit to patients.

## 5 Conclusions and perspectives

In recent years, anti-PD-1/PD-L1 therapy for NSCLC has made impressive progress. Compared with conventional chemotherapy, anti-PD-1/PD-L1 therapy has shown better clinical efficacy in patients with advanced NSCLC. Therapeutic strategies that utilize the gut microbiota in combination with ICIs, including probiotics, prebiotics intake, fecal microbiota transplantation(FMT) and other dietary interventions, represent new possibilities in effective adjuvant for anti-PD-1/PD-L1 therapy. Further understanding of the synergistic mechanism between anti-PD-1/PD-L1 treatment and gut microbiota is expected to develop more effective combined treatment strategies for anti-PD-1/PD-L1 treatment and promote the development of precision medicine treatment strategies. However, there are still many issues associated with this combination therapy.

Firstly, studies have shown that FMT has a certain auxiliary effect on tumor immunotherapy (27, 54, 92). However, the potential risks of disease transmission between donors and recipients, patient acceptance, undesirable outcomes, and uncertainties regarding effects on the immune system of recipients underscore the critical need for large-sample randomized controlled studies to comprehensively assess the effectiveness of FMT (93, 94). While significant progress has been made in understanding the human gut’s bacterial population in recent years, there remains limited knowledge about the composition, pathogenicity, and function of gut bacteria. Additionally, another uncertainty of FMT is the highly dynamic composition of live microbiota, which is sensitive to external factors such as diet and drugs (95). Therefore, future research should focus on identifying the gut microbiota, defining their functions, and further manipulating the gut microbiota more precisely. We will expect to personalize FMT for different patients based on different hosts and disease genotypes.

In addition, tools for diagnosing the gut microbiota in patients with cancer still need to be refined, and more research is needed to develop new diagnostic tools based on the gut microbiota of patients with cancer to predict the response and resistance to ICIs. Third, although many studies have provided an abundance of evidence, there is still controversy related to some conclusions. The design of additional experiments and tighter research controls are needed to support each conclusion. Finally, Some microbiota and diet-assisted therapies are still in their nascent stage. Researchers are still working hard to design robust clinical trials with the aim of overcoming issue related to variability and biases to verify and utilize the causal relationship between gut microbiota and the efficacy and safety of immunotherapy. In this way, a more rigorous, safe, and reliable, adjuvant treatment strategy in anti-PD-1/PD-L1 therapy for NSCLC can be developed. FMT and dietary intervention are expected to become powerful auxiliary tools in the anti-PD-1/PD-L1 therapy of NSCLC, raising the standard of lung cancer treatment to new heights.

## Author contributions

YX: Writing – original draft. CGL: Writing – original draft. DZ: Writing – review & editing. JC: Writing – review & editing.
